# Amyloid-related imaging abnormalities (ARIA) in Alzheimer’s disease: from pathophysiology to individualized risk assessment

**DOI:** 10.1186/s13195-026-02022-7

**Published:** 2026-03-28

**Authors:** Jonathan Merkel, Robert Perneczky, Frank Jessen, Lutz Frölich, Olav Jansen, Sönke Peters, Daniela Berg, Jörg B. Schulz, Thorsten Bartsch

**Affiliations:** 1 Department of Neurology, University Hospital Schleswig-Holstein, 24105 Kiel, Germany; 2 Department of Psychiatry and Psychotherapy, LMU Munich, University Hospital, Nussbaumstr. 7, Munich, 80336 Germany; 3 German Center for Neurodegenerative Diseases (DZNE) Munich, Marchioninistr. 15, 81377 Munich, Germany; 4 Munich Cluster for Systems Neurology (SyNergy), Marchioninistr. 15, 81377 Munich, Germany; 5 Ageing Epidemiology (AGE) Research Unit, School of Public Health, Imperial College London, Fulham Palace Road, London, W6 8RP UK; 6 Sheffield Institute for Translational Neuroscience (SITraN), University of Sheffield, 385a Glossop Road, Sheffield, S10 2HQ UK; 7 Department of Psychiatry and Psychotherapy, Medical Faculty, University of Cologne, Cologne, Germany; 8 German Center for Neurodegenerative Diseases (DZNE), Bonn/Cologne, Germany; 9 Excellence Cluster on Cellular Stress Responses in Aging-Associated Diseases (CECAD), University of Cologne, Cologne, Germany; 10 Department of Geriatric Psychiatry Central Institute of Mental Health, Mannheim, Germany; 11 Clinic of Radiology and Neuroradiology, University Hospital Schleswig- Holstein, 24105 Kiel, Germany; 12 Department of Neurology, RWTH Aachen University, Aachen, Germany; 13 JARA-BRAIN Institute Molecular Neuroscience and Neuroimaging, Forschungszentrum Jülich GmbH and RWTH Aachen University, Aachen, Germany

**Keywords:** Amyloid-related imaging abnormalities, ARIA, Amyloid-targeting therapies, Alzheimer’s disease, Dementia, Cerebral amyloid angiopathy, Neurodegeneration, Neuroinflammation

## Abstract

Monoclonal antibodies targeting amyloid-β are the first approved disease-modifying treatment for Alzheimer’s disease. While amyloid-targeting therapies mitigate the progression of cognitive decline in early-stage Alzheimer’s disease, they are associated with amyloid-related imaging abnormalities (ARIA), an imaging phenomenon presenting as cerebral edema/effusion and/or hemorrhage. Redistribution of parenchymal amyloid-β to perivascular drainage pathways and direct antibody-amyloid interactions within the cerebral vasculature are considered key players in ARIA pathophysiology by promoting inflammation and vascular disruption, thus mirroring hallmarks of inflammatory cerebral amyloid angiopathy. Although ARIA is commonly regarded as an undesired side effect of amyloid-targeting therapies, its association with amyloid-β clearance from the brain opens up the possibility of an alternative interpretation as a physiological reaction to target engagement of anti-amyloid antibodies.

Understanding risk factors that promote the occurrence of ARIA and its transformation from asymptomatic imaging phenomenon to its serious and severe form are of great importance to clinical practice. ARIA risk and severity are influenced by apolipoprotein E4 status, microvascular damage, and cerebral amyloid angiopathy, but may be further modulated by antibody binding preferences and comorbidities such as arterial hypertension and ischemic strokes. Identifying individual risk profiles based on deeper insights into pathophysiological pathways may improve patient safety and lead to personalized treatment concepts in Alzheimer’s disease.

In this review, we provide a comprehensive summary of ARIA pathophysiology, highlight important risk factors and discuss their relevance in clinical risk management.

**Supplementary Information:**

The online version contains supplementary material available at 10.1186/s13195-026-02022-7.

## Background

Alzheimer’s disease (AD) is a progressive neurodegenerative disease and the most common cause of dementia [1]. With the “amyloid cascade hypothesis” as a leading theory of AD pathogenesis, the development of monoclonal antibodies targeting amyloid-β (Aβ) has led to the first disease-modifying therapies of early-stage AD [2]. While Aβ immunotherapy reduces Aβ burden and slows down the rate of clinical disease progression, exposure to these antibodies is associated with amyloid-related imaging abnormalities (ARIA) [3, 4]. Approval of the anti-Aβ antibodies lecanemab and donanemab by multiple drug regulatory agencies including the U.S. Food and Drug Administration (FDA), the European Medicines Agency (EMA), and the Medicines and Healthcare products Regulatory Agency (MHRA) highlights the importance of increasing patient safety by transferring our knowledge on ARIA into clinical practice. This review connects pathophysiological concepts of ARIA with relevant risk factors and discusses their clinical implications, providing an up-to-date overview of ARIA and future directions towards individualized risk assessment and targeted preventative and therapeutic strategies.

### Search strategy and selection criteria

Articles published in English between January 2000 and December 2025 were used as references for this review and searched via PubMed, using the search terms “amyloid-related imaging abnormalities”, “cerebral amyloid angiopathy”, “lecanemab”, “donanemab”, “aducanumab”, “gantenerumab”, and “risk factors AND amyloid-related imaging abnormalities”. The final reference list was generated based on relevance to the topics covered in this review. The prescribing information of lecanemab and donanemab were obtained from the FDA, EMA, and MHRA. The online sources referring to trontinemab were searched via Google using the search terms “trontinemab AND alzforum”.

Data from clinical trials used to illustrate Fig. 3 were included based on public availability of ARIA incidence and amyloid positron emission tomography (PET) status in centiloids pre- and post-treatment. Since the aducanumab trials (ENGAGE, EMERGE) reported separate incidences of microhemorrhage and superficial siderosis instead of total ARIA-H, the incidence of ARIA-H was taken from a secondary source [5], summarizing ARIA-H rates of EMERGE and ENGAGE for the low and high dosing regimens. Consequently, PET centiloid changes were averaged from the EMERGE and ENGAGE trials for high and low dose. PET centiloid changes of the lecanemab phase 2 trial (BAN 2401 − 201) were estimated from graphs published by McDade et al. [6] due to lacking public availability of the original values. All other data were obtained from the original clinical trials (refer to supplement). All graphs were created using GraphPad Prism. MR images of ARIA were reprinted from Barakos et al. [7] under the terms of the Creative Commons CC-BY license.

### ARIA definition and subtypes

**Fig. 1:**
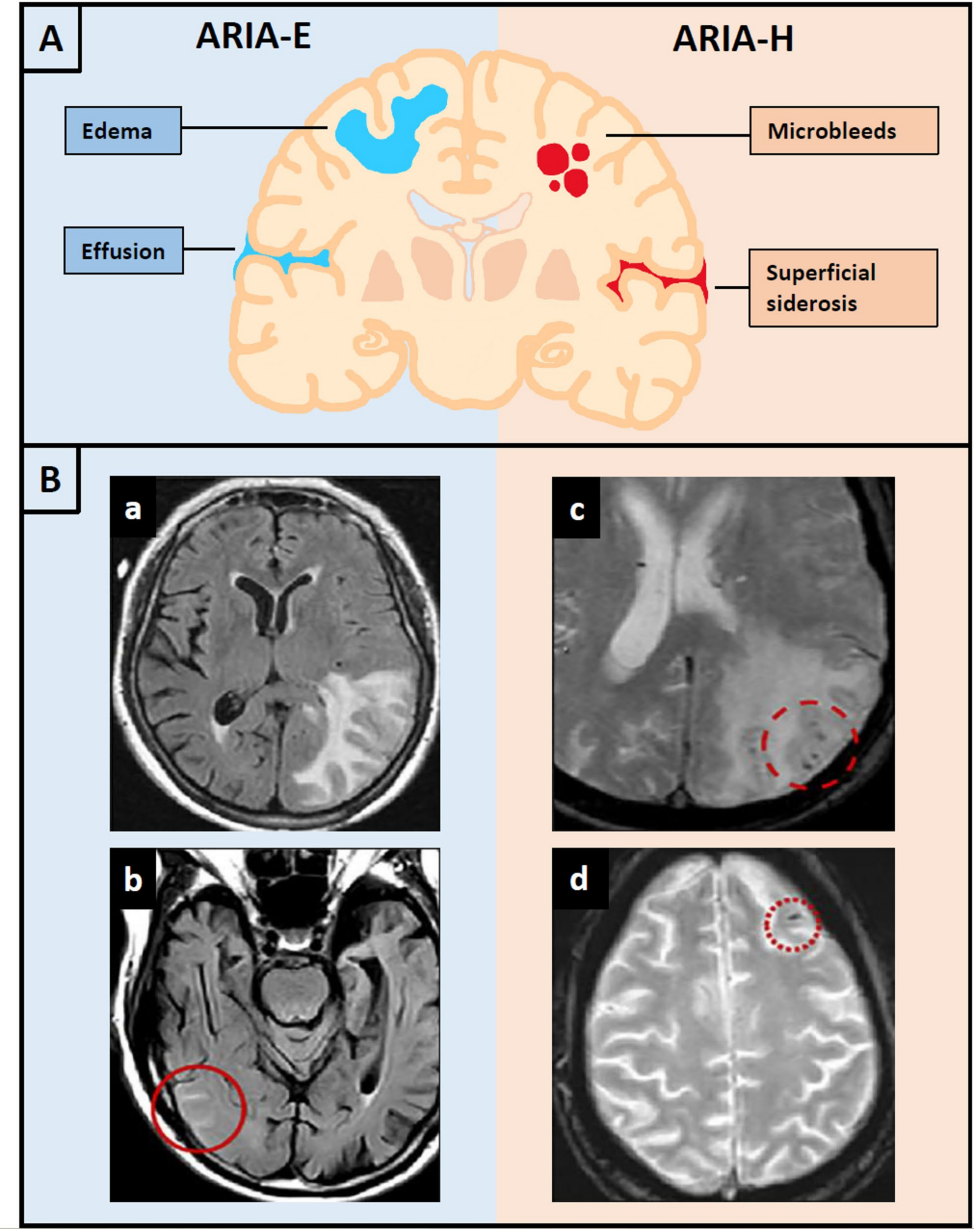
**A** Illustration of ARIA subtypes. ARIA-E occurs as interstitial edema and/or sulcal effusion. ARIA-H is characterized by parenchymal microbleeds and/or superficial siderosis. Most ARIA-H is associated with concomitant ARIA-E. **B** Representative MR images of ARIA-E with vasogenic edema (a) and sulcal effusion (b) on axial FLAIR sequences. Representative images of ARIA-H with microhemorrhage (c) and superficial siderosis (d) on axial T2* GRE (reprinted from Barakos et al. [7] under the terms of the creative commons CC-BY license)

The term ARIA refers to a spectrum of magnetic resonance imaging (MRI) phenomena which can be detected over the course of Aβ immunotherapy [8]. The first subtype, ARIA with edema/effusion (ARIA-E), is attributed to proteinaceous fluid extravasation into the brain parenchyma and/or leptomeningeal compartment, resulting in interstitial edema and/or sulcal effusion. The second subtype, ARIA with hemorrhage (ARIA-H), is characterized by parenchymal microbleeds and/or superficial siderosis (Fig. 1A) [7, 8]. ARIA-E occurs in 12.6–24.4% and ARIA-H in 16.9–31.3% of lecanemab-/donanemab-treated patients [9, 10]. The two subtypes frequently coincide – in fact, 91% of ARIA-H cases caused by lecanemab were associated with concomitant ARIA-E [3]. Although ARIA has been attributed to Aβ immunotherapy, it also occurs as a natural part of AD with ARIA-E rates of 1.7–1.9% and ARIA-H rates of 8.9–13.0% in placebo-treated groups [9, 10].

On MRI (Fig. 1B), ARIA-E is visualized as T2 or fluid attenuation inversion recovery (FLAIR) hyperintensities, whereas ARIA-H can be detected as signal voids on T2* gradient recalled echo (GRE) or more sensitively on susceptibility-weighted imaging (SWI) [7, 8]. Neither ARIA subtype is associated with restricted diffusion, which is crucial to differentiate ARIA from acute cerebral ischemia [8]. While ARIA-E usually resolves without any sequelae, ARIA-H tends to stabilize over time but results in permanent hemosiderin deposits in the brain [11].

### Symptoms and severity of ARIA

Most cases of ARIA are asymptomatic and only detected on MRI [7]. In the lecanemab and donanemab phase 3 trials, 2.8–5.8% of ARIA-E and 1.0–1.2% of ARIA-H were reported as symptomatic [9, 10]. Symptoms are usually mild and unspecific such as headache (10.4–13.5%), confusion (3.9–5.4%), dizziness (1.4–1.7%), nausea (0.5–1.7%), gait disturbance (1.3–2.4%), and vision changes [10, 12]. Although reported in only 0.7–1.6% of cases, serious symptoms in the context of ARIA can occur, including seizures, encephalopathy, and focal neurological deficits [9, 10, 12]. ARIA can mirror neurological conditions such as progression of AD dementia, stroke, and posterior reversible encephalopathy syndrome [13, 14], stressing the importance of clinical and radiological expertise when treating AD patients with Aβ-targeting therapies. While rare, death has been associated with ARIA, with 3 out of 17 deaths related to ARIA in donanemab-treated patients compared to 12 total deaths in the placebo group, none of which were associated with ARIA [10], yet exposure-adjusted mortality rates were similar between lecanemab and placebo (0.0069 per participant per year vs. 0.0065 per participant per year) [9]. Some deaths related to ARIA were complicated by additional circumstances (e.g. administration of a thrombolytic agent, underlying cerebral amyloid angiopathy) [9, 15, 16].

ARIA severity is graded by using MRI classification criteria (Table 1) [12, 13]. ARIA-E and ARIA-H graded as severe on MRI occur in 2.1–11.5% and 3.5–6.4% of cases, respectively [9, 10]. Since MRI findings do not necessarily correlate with clinical presentation, both clinical and MRI features need to be considered when monitoring and managing ARIA [12, 13].

**Table 1:** MRI classification criteria of ARIA severity [12, 14]

**ARIA subtype**	**Severity**		
	**Mild**	**Moderate**	**Severe**
ARIA-E			
Size of FLAIR hyperintensity (sulcal, cortical/subcortical)	<5 cm in one location	5 to 10 cm in one location or <10 cm in multiple locations	>10 cm in one or multiple locations with associated gyral swelling and sulcal effacement
ARIA-H			
No. of new incident microhemorrhages	1-4	5-9	≥10
No. of focal areas of superficial siderosis	1	2	≥3

### Pathophysiological concepts of ARIA

#### Vascular amyloid overload hypothesis

Evidence suggests that ARIA is caused by proinflammatory antibody-Aβ interactions within and around the cerebral vasculature due to reallocation of parenchymal Aβ to cerebral blood vessels during the Aβ clearance process. Aβ is thought to be removed from the brain by transportation across the blood-brain barrier and via perivascular drainage pathways [17]. Levels of Aβ_1–42_ in the cerebrospinal fluid (CSF) have been reported to increase with Aβ immunotherapy [18], indicating breakdown of Aβ plaques and efflux of soluble Aβ from the brain into the CSF via the perivascular system. Antibody-mediated mobilization of plaque-bound Aβ and its transportation along perivascular pathways may increase vascular Aβ load and elicit an immune response against vessel walls and perivascular structures [8, 19]. As a second mechanism, Aβ-targeting antibodies seem to interact with vascular Aβ directly, thereby driving inflammation and compromising vascular integrity, as evidenced by studies showing that anti-Aβ antibodies bind vascular Aβ in vitro and promote vascular disruption in a mouse model of AD [20, 21]. Neuropathological analyses of human ARIA cases also identified perivascular inflammation and vessel wall degeneration as hallmarks of ARIA [15, 22]. Increased vascular permeability eventually leads to extravasation of plasma components and erythrocytes, causing edema/effusion (ARIA-E) and hemorrhage (ARIA-H) [8].

#### Commonalities between ARIA and CAA

Cerebral amyloid angiopathy (CAA) coincides with AD in up to 80% of cases and is characterized by Aβ deposition in the cerebral vasculature, resulting in fragile and dysfunctional blood vessels [17, 23]. MRI criteria of CAA include lobar microbleeds and superficial siderosis [23, 24], hence resembling ARIA-H, while an inflammatory subtype of CAA (inflammatory CAA, iCAA) shares key features with ARIA-E on MRI and histopathological examination (Table 2). iCAA is assumed to be an autoimmune response against vascular Aβ and can be distinguished into two subtypes: CAA-related inflammation (CAA-ri), defined as a non-angiodestructive, mostly lymphocytic, perivascular inflammatory process, and amyloid-β related angiitis (ABRA), characterized by angiodestructive, often granulomatous inflammation of Aβ-positive vessel walls [23]. On MRI, both iCAA and ARIA-E show leptomeningeal enhancement and multifocal edema [23, 25]. On autopsy, ARIA-E presents with perivascular inflammation by macrophages/microglia and T lymphocytes, complement activation, transmural infiltration by multinucleated giant cells, and fibrinoid degeneration of Aβ-positive vessels, bearing close resemblance to the hallmarks of ABRA [15, 22, 23]. Intrinsic anti-Aβ antibodies can be detected in the CSF of CAA-ri and ABRA patients, pointing to an antibody-mediated disease process that resembles the assumed pathophysiology of ARIA [23, 24]. Due to these commonalities, ARIA-E is widely considered an iatrogenic subtype of iCAA [17].

**Table 2:** Comparison of cerebral amyloid angiopathy (CAA) and ARIA subtypes

Name	Definition	Pathology + assumed pathophysiology	Possible MRI findings
Cerebral amyloid angiopathy (CAA)	Cerebral vasculopathy caused by Aß deposition in the walls of leptomeningeal and cortical blood vessels [17, 23]	Transmural Aß deposition leads to fragile vessel walls, causing cerebral hemorrhage without inflammation [17]	Lobar microbleeds, sulcal siderosis, macrohemorrhage [23, 24], white matter changes [17]
Inflammatory CAA (iCAA)	Inflammation triggered by an autoimmune response to vascular Aß deposits of leptomeningeal and cortical blood vessels [23]	Subtypes [23]:1. CAA-related inflammation (CAA-ri): Perivascular, mostly lymphocytic inflammation without angiodestruction 2. Amyloid-beta related angiitis (ABRA): Transmural, granulomatous inflammation with angiodestruction, i.e. vasculitis	Multifocal edema, sulcal effusion, microbleeds, sulcal siderosis [23]
Amyloid-related imaging abnormalities (ARIA)	MRI abnormalities associated with Aß immunotherapy in patients with Alzheimer's disease [8]		
ARIA with edema (ARIA-E)	Vasogenic edema and/or leptomeningeal effusion in the context of Aß immunotherapy [8]	Perivascular and some transmural inflammation against Aß deposits in the cerebral vasculature elicited by Aß immunotherapy [15, 17]	Multifocal edema, sulcal effusion [25]
ARIA with hemorrhage (ARIA-H)	Cerebral hemorrhage in the context of Aß immunotherapy, often concomitant with ARIA-E [8]	(Peri-)vascular inflammation due to Aß immunotherapy leads to extravasation of erythrocytes into the brain, causing cerebral hemorrhage [8]	Microbleeds, sulcal siderosis [25]

#### Time course of ARIA

**Fig. 2:**
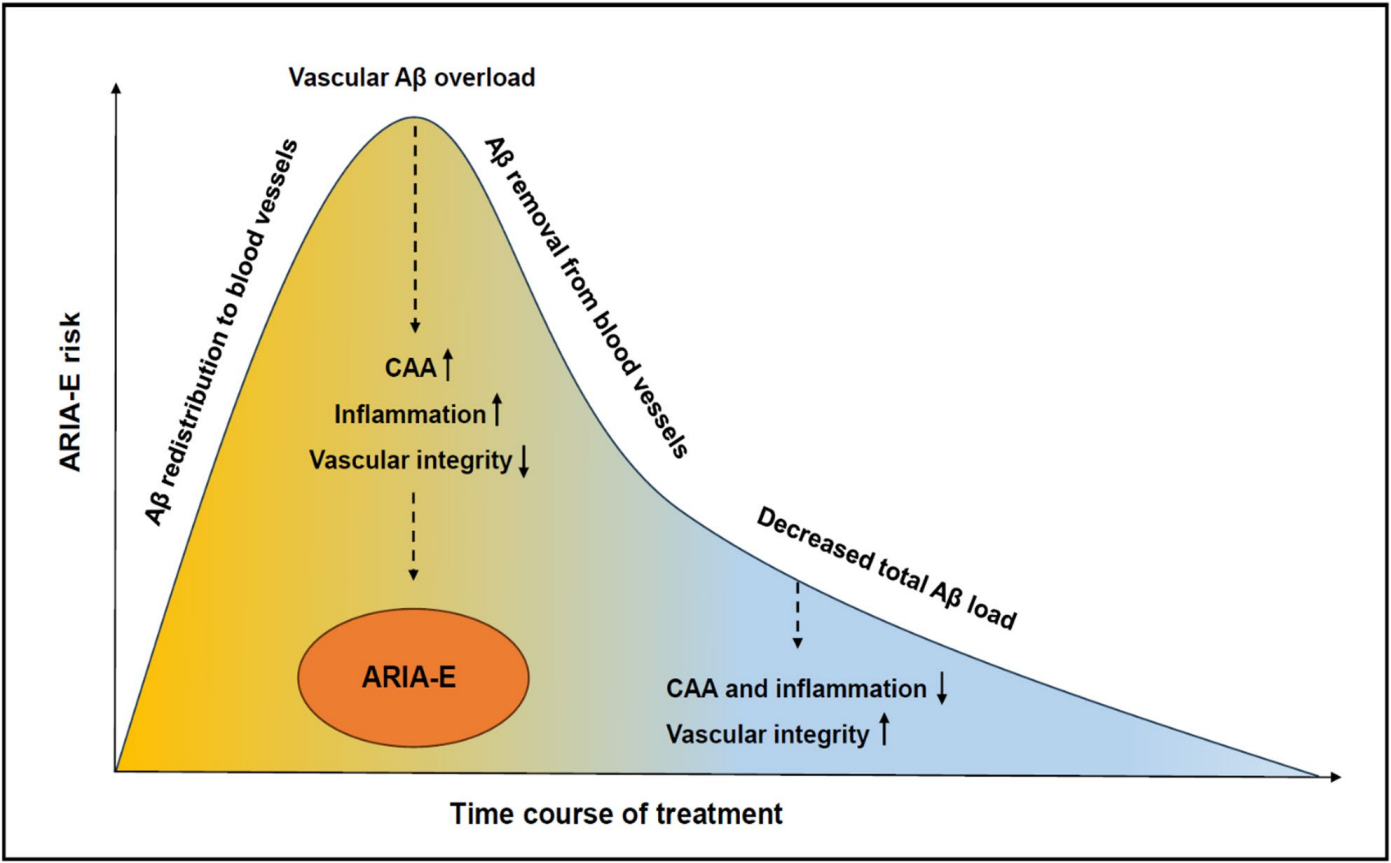
Illustration of the relationship between ARIA-E risk over time and the putative pathomechanisms of ARIA with redistribution of parenchymal Aβ to cerebral blood vessels leading to transient vascular Aβ overload, inflammation, and increased vascular permeability

ARIA-E generally occurs early after exposure to Aβ immunotherapy, with 58–70% of ARIA-E events detected within the first three months and 92% within the first six months of treatment [3, 4, 10]. This peak probably corresponds to the phase of Aβ mobilization to blood vessels and subsequent drainage overload. The risk of ARIA-E decreases with ongoing treatment as Aβ is removed from the brain and cerebral vasculature (Fig. 2) [7].

ARIA-E is a mostly self-limiting process, as ~ 80% of cases associated with lecanemab resolved within four months after detection and 96% of donanemab-associated ARIA-E resolved within a mean time of two months [3, 9, 10]. Spontaneous resolution of ARIA-E may be explained by the temporary impairment of homeostatic Aβ clearance mechanisms. As perivascular clearance pathways catch up with increased demand, Aβ is removed from the cerebral vasculature, inflammation may subside, and vascular integrity be restored [7]. This hypothesis is supported by a mouse model of AD immunized with an anti-Aβ antibody showing transiently increased cerebral microbleeds and capillary Aβ burden but restored long-term vascular integrity associated with time-dependent clearance of vascular and parenchymal Aβ [7].

The association between incidence and time course of treatment is less apparent with ARIA-H and requires separate examination of ARIA-H without ARIA-E, i.e. isolated ARIA-H (9% of cases), versus the more common ARIA-H concurrent with ARIA-E (91% of cases) [9]. While the timing of concurrent ARIA-H resembles that of ARIA-E, the rate and timing of isolated ARIA-H in lecanemab-treated patients has been found to be that of placebo [9]. The phenomenon described as isolated ARIA-H may thus not be linked to Aβ immunotherapy but result from accumulation of microhemorrhages in the brain due to concomitant hypertension, CAA, or AD as part of the disease process.

#### Relationship between ARIA and binding profiles of anti-Aβ antibodies

**Fig. 3:**
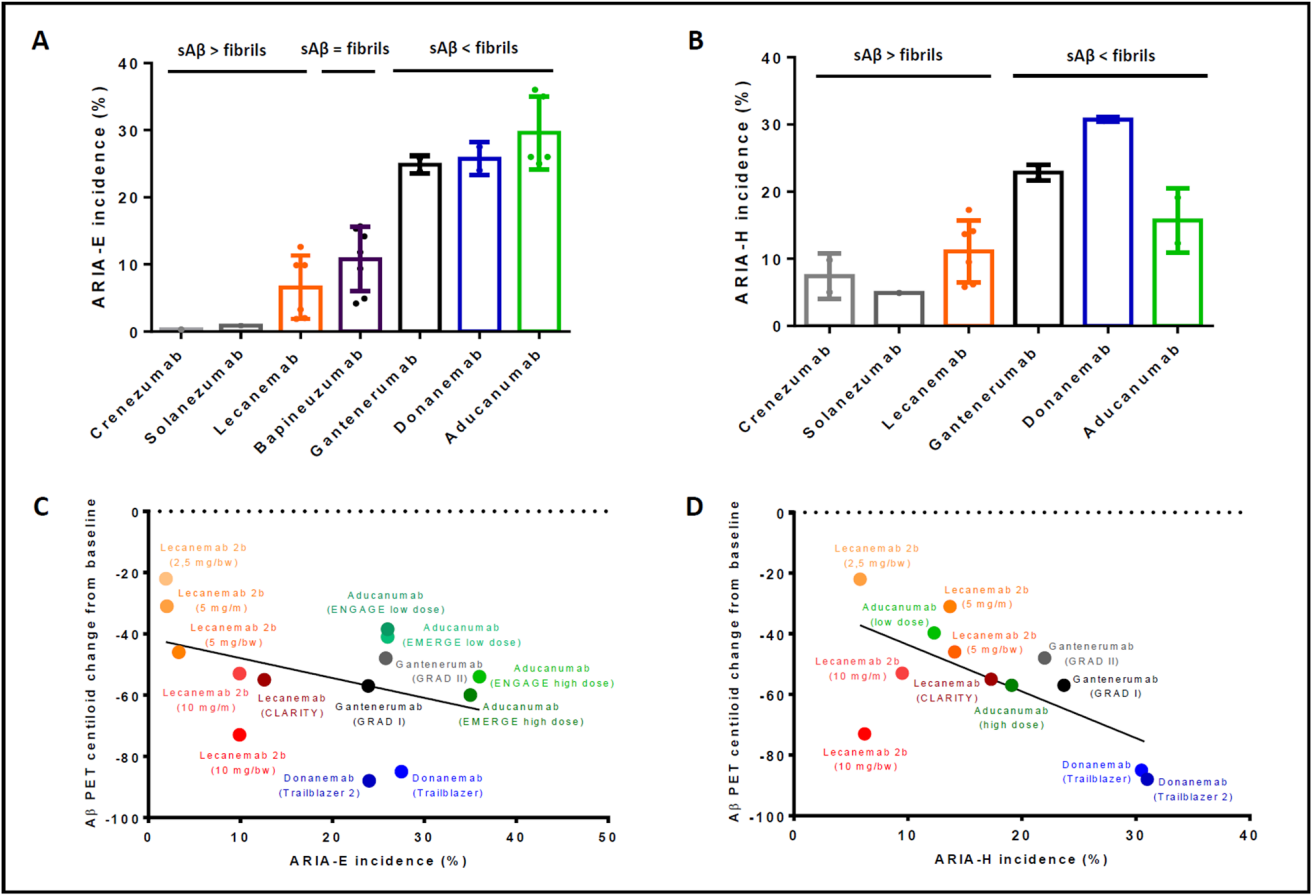
Monoclonal antibodies with a binding preference for fibrillary Aβ (fibrils) over soluble Aβ (sAβ) are associated with higher rates of ARIA-E (**A**) and ARIA-H (**B**). Illustrative graphs showing the association between Aβ plaque clearance in PET centiloid change from baseline and incidence of ARIA-E (**C**) and ARIA-H (**D**) across multiple phase 2 and 3 trials with different antibodies and dosages. Figure 3B and D show combined rates of concurrent and isolated ARIA-H

The risk of ARIA varies depending on the anti-Aβ antibody used. Antibodies targeting the N-terminus of Aβ (e.g. aducanumab, donanemab) cause more ARIA than those binding to the C-terminus or mid-domain (e.g. solanezumab, crenezumab). Binding preferences to different Aβ aggregate states, i.e. soluble versus fibrillary Aβ, also influence ARIA risk (Fig. 3A and B). Antibodies targeting fibrillary Aβ (e.g. aducanumab, donanemab, gantenerumab) are associated with higher ARIA rates than those with a binding preference for protofibrils (lecanemab) or mono-/oligomers (solanezumab, crenezumab) but also demonstrate greater efficacy on plaque removal [2, 7, 26], while antibodies binding soluble Aβ, with the exception of lecanemab, failed to significantly reduce Aβ pathology in clinical trials [3, 27, 28]. Since ARIA is potentially related to Aβ redistribution and removal, ARIA-E rates as low as 0.9% (solanezumab) and 0.3% (crenezumab) may be due to the failure of these two antibodies to clear Aβ from the brain [19, 27, 28]. Additionally, antibodies with higher ARIA-E rates show increased binding to CAA fibrils, supporting the hypothesis that direct interactions between anti-Aβ antibodies and vascular Aβ drive ARIA pathophysiology [21].

#### Relationship between ARIA and dosage of anti-Aβ antibodies

ARIA-E risk increases with higher doses of the respective anti-Aβ antibody used [7]. It remains to be determined whether the peak concentration of the antibody or the cumulative dose are responsible for this phenomenon [17]. Dose titration has been shown to reduce the risk of ARIA-E in a modified dosing regimen of donanemab without affecting Aβ reduction [29], suggesting that a slower increase in serum antibody concentration may reduce ARIA risk by decreased binding to vascular Aβ and a more gradual release of Aβ from plaques. Conversely, the maximum serum concentration of lecanemab has been positively correlated with ARIA-E probability [30], suggesting that the antibody’s peak serum concentration may be involved in promoting ARIA and pointing to a complex relationship between antibody dosage, pharmacodynamics/-kinetics, and ARIA.

#### Relationship between ARIA and Aβ clearance

According to the vascular amyloid overload hypothesis, Aβ redistribution from plaques to perivascular clearance pathways may contribute to ARIA; however, the relationship between ARIA and Aβ clearance has not been thoroughly investigated. Herein, Aβ clearance efficacy indicated as PET centiloid change from baseline and reported ARIA incidence of multiple antibodies across phase 2 and 3 trials are summarized (Fig. 3C and D) [3, 4, 18, 31–33]. Individual values used for Fig. 3 and their respective sources are shown in supplement 1.

Although preliminary and not comparable on a statistical level due to different study designs, the overall trend points to higher incidence of ARIA-E and ARIA-H with increased Aβ clearance capacity across different antibodies and dosages, suggesting a positive correlation between Aβ clearance and ARIA incidence. There was no significant difference in the percentage of apolipoprotein E4 carriers across antibodies (data not shown). Since ARIA pathophysiology is thought to be linked to Aβ redistribution and clearance, a desired effect by Aβ immunotherapy, it may be considered a physiological reaction to the Aβ removing process and a reflection of target engagement of Aβ-lowering therapies instead of a pathological side effect in the classical sense. Importantly, occurrence of ARIA does not impact cognitive outcome, as patients that experienced ARIA benefited equally well from treatment than those without ARIA [9]. While especially mild and asymptomatic ARIA may therefore be considered a natural, albeit not predetermined consequence of antibody-mediated Aβ clearance from the AD brain, risk factors have been identified that increase ARIA frequency and severity.

### Risk factors of ARIA and their clinical implications

Prior to Aβ immunotherapy, it is crucial to identify risk factors that may impact ARIA occurrence and severity. In the following section, ARIA risk factors and their clinical implications are summarized in accordance with the prescribing information of lecanemab and donanemab and their appropriate use recommendations [12–14, 34].

#### Apolipoprotein E genotype

The genetic risk for late-onset AD is most strongly determined by the apolipoprotein E (APOE) gene which influences major pathogenic cascades in AD [35–37]. In humans, three isoforms of the APOE gene exist: ε2 (APOE2), ε3 (APOE3), and ε4 (APOE4). These isoforms predispose to AD in different manners, with APOE3 as the neutral benchmark of AD risk. APOE2 reduces the risk of AD by almost 50%, while APOE4 is the strongest known genetic risk factor for late-onset AD, with heterozygous APOE4 carriers exhibiting a three- to fourfold and homozygous carriers a 12-15-fold increase in AD risk compared to APOE3 [1, 38].

Clinical trials have consistently reported that APOE4 increases ARIA risk in a dose-dependent manner [9, 10, 31, 33]. During treatment with lecanemab, participants heterozygous for the APOE4 allele had an almost two-fold increase (11.6%) and homozygous carriers (APOE4/4) a five-fold increase (34.5%) of ARIA-E compared to non-carriers (6.5%). For ARIA-H, incidence was doubled in APOE4-positive participants (21.4%) compared to their APOE4-negative counterparts (11.9%). APOE4 carriers are also more prone to symptomatic and severe ARIA, with a rate of symptomatic ARIA of 1.6–2.6% in APOE4 non-carriers compared to 6.1–11.2% in APOE4/4 participants [9, 10].

APOE4 may predispose the brain to ARIA by multiple mechanisms. While the major physiological function of APOE is cholesterol and lipid transport in the periphery and the brain, it also plays an isoform-specific role in neurovascular stability [38, 39]. APOE4 can compromise the integrity of the blood-brain barrier [40–42], which may contribute to ARIA by increasing vascular permeability. APOE4 has also been implicated in neuroinflammation and a dysregulated immune response [43–45], potentially predisposing carriers to more frequent, severe, and symptomatic ARIA. Furthermore, APOE4 promotes Aβ accumulation in the brain and cerebral vasculature, leading to more pronounced AD pathology and CAA [37, 39], which may fuel the development of ARIA due to higher parenchymal and vascular Aβ burden.

The use of lecanemab and donanemab in the United States (US) has been approved regardless of APOE genotype, but genotyping prior to treatment is strongly recommended to assess ARIA risk, enhance clinical vigilance, and intensify MRI monitoring in APOE4 carriers [12, 13]. In the European Union (EU) and United Kingdom (UK), lecanemab and donanemab have been approved only for APOE4 non-carriers and APOE4 heterozygotes, making APOE genotyping a prerequisite for the initiation of Aβ immunotherapy in these countries [14, 34].

#### Baseline cerebral hemorrhage and CAA

Baseline microhemorrhages and superficial siderosis are established risk factors of ARIA and can occur within the context of CAA [9, 10]. With regard to the parallels between ARIA and iCAA, the presence of vascular Aβ deposits may be a precondition for ARIA to develop. Microbleeds not attributable to CAA, e.g. due to hypertensive vasculopathy, may also contribute to ARIA as they are indicators of microvascular damage [46].

While not listed as a contraindication by the FDA, patients with evidence of cerebral hemorrhage (> 4 microhemorrhages, ≥ 1 macrohemorrhage, ≥ 1 superficial siderosis), or evidence of ABRA/CAA-ri on baseline MRI should be excluded from treatment with lecanemab and donanemab in the US [12, 13]. In the EU and UK, aforementioned criteria of cerebral hemorrhage or CAA are contraindications to lecanemab and donanemab, while it is additionally advised against lecanemab if baseline MRI shows evidence of ≥ 2 lobar microbleeds, taking into account the Boston Criteria v1.5 of probable CAA [14, 34].

#### White matter changes and prior strokes

White matter hyperintensities on baseline MRI equivalent to a Fazekas score of 3 are associated with higher ARIA risk [9,10]. Similarly to microbleeds, white matter changes constitute a sign of microvascular damage and CAA [17, 46], which may increase vulnerability to ARIA. The association between ARIA and cerebrovascular events involving larger vessels is less clear. Since patients with evidence of a recent stroke or transient ischemic attack were excluded from clinical trials, empirical data are lacking. Hypothetically, cytotoxic edema and vascular injury due to recent ischemia could predispose to ARIA [11].

While not listed as an explicit contraindication by the FDA, US recommendations advise against treatment with lecanemab/donanemab if baseline MRI shows evidence of extensive white matter changes, a major territorial stroke or > 2 lacunar infarcts [12, 13, 34, 47]. In the EU and UK, similar recommendations apply [14, 34]. Additionally, severe white matter changes are listed as a contraindication to donanemab by the EMA and MHRA.

#### Arterial hypertension

Poorly controlled hypertension has been identified as a risk factor of ARIA in the context of donanemab [10], whereas no association between arterial hypertension and ARIA has been found in the phase 3 lecanemab study [9]. Poorly controlled hypertension may compromise the integrity of small blood vessels and contribute to ARIA.

It is recommended that only patients with well controlled hypertension receive donanemab in the US [13], and donanemab is contraindicated in patients with poorly controlled hypertension in the EU.

#### Antithrombotic medication

While the concomitant use of antithrombotic medication (i.e. antiplatelets and anticoagulants) has not been shown to significantly impact ARIA risk in the phase 3 trials of lecanemab and donanemab [9, 10], patients on lecanemab co-medicated with anticoagulants (e.g. vitamin K antagonists, novel oral anticoagulants) experienced a 2.7% incidence of cerebral macrohemorrhage, a rare but severe complication of Aβ immunotherapy, compared to 0.6% in lecanemab-treated patients without anticoagulants [9]. However, donanemab combined with anticoagulants does not appear to increase the risk of macrohemorrhage [13]. Other risk factors such as preexisting CAA and administration of thrombolytic agents for the treatment of stroke-like symptoms retrospectively identified as ARIA may have contributed to the occurrence of macrohemorrhage in some cases [10, 13, 16]. Due to overall small case numbers, no definite conclusions can be drawn at this point regarding the concomitant use of anticoagulants and the risk of cerebral macrohemorrhage [9].

Comedication with anticoagulants is not listed as a contraindication to lecanemab/donanemab by the FDA, but it is generally advised against the concomitant use of anticoagulants until more safety data emerge [12, 13, 47]. In the EU and UK, it is contraindicated to administer lecanemab/donanemab to patients on anticoagulants [14, 34]. Consequently, Aβ immunotherapy should be discontinued if the need of long-term anticoagulation arises [13, 14, 34]. In certain cases, alternatives to anticoagulation such as a left atrial appendage occluder may be discussed, which is, however, not recommended as a routine procedure [34]. Close cooperation between cardiologists, emergency physicians and neurologists is required if patients on Aβ immunotherapy present with urgent medical conditions that require immediate anticoagulation. If anticoagulation cannot be avoided, increased clinical vigilance and low-threshold MRI availability are necessary to detect early signs of ARIA or cerebral macrohemorrhage. The use of thrombolytics is generally not recommended in patients on Aβ immunotherapy due to concerns about increased risk of cerebral hemorrhage and should only be applied on a case-by-case basis in serious situations with no alternative treatment options and after excluding ARIA on MRI, especially if patients present with stroke-like symptoms [13, 14, 34].

Antiplatelet medication (e.g. aspirin, clopidogrel) may be continued while on Aβ immunotherapy since it does not appear to significantly increase the risk of ARIA and cerebral macrohemorrhage, although analysis regarding macrohemorrhage is statistically underpowered [10, 12, 13, 34]. Dual antiplatelet therapy has not been found to significantly impact ARIA risk but should be used with caution due to the lack of larger safety datasets [9, 10, 14, 34].

### Monitoring and management of ARIA

#### ARIA monitoring

Clinical evaluation for ARIA symptoms is recommended during each visit and before each antibody infusion, with heightened clinical vigilance over the first 14 weeks due to a peak in ARIA early after treatment initiation. Routine MRI scans should be obtained as recommended, consisting of a minimum standardized protocol of FLAIR, T2* GRE/SWI, and diffusion-weighted imaging [12–14, 34, 48].

#### ARIA management

**Fig. 4:**
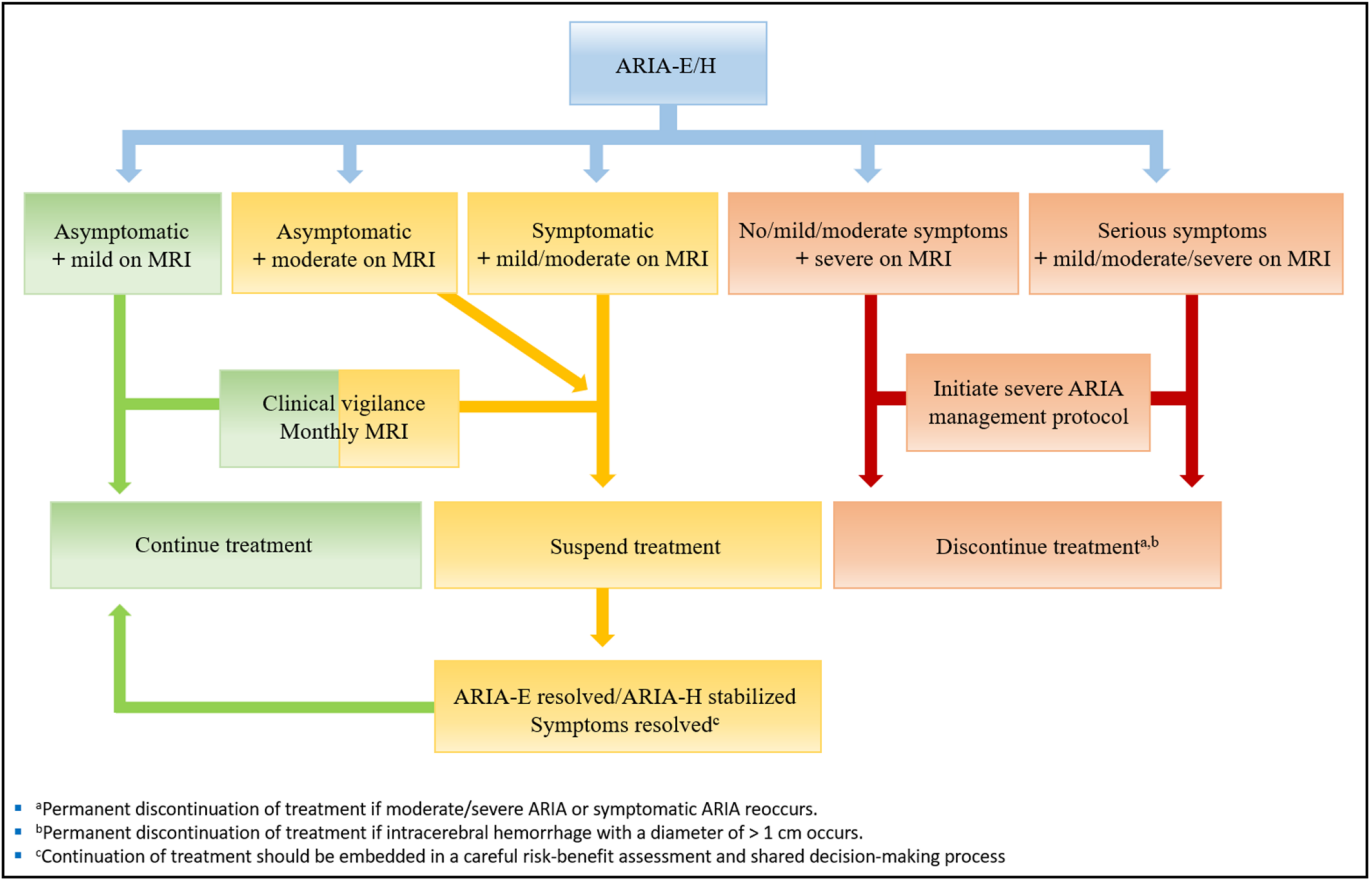
Flowchart visualizing clinical ARIA management based on current appropriate use recommendations [12–14, 34]

Management of ARIA depends on clinical and radiographic severity (Fig. 4). In cases of mild asymptomatic ARIA, treatment may be continued, and monthly MRIs are required until ARIA-E resolves or ARIA-H stabilizes. In patients with symptomatic ARIA or moderate ARIA on MRI, treatment should be suspended, and monthly MRIs be obtained [12–14, 34, 48]. Once symptoms have subsided and ARIA has resolved/stabilized, treatment may be resumed after a careful risk-benefit assessment [12–14, 34]. The outcome of mild and moderate ARIA is generally favorable, and ~ 80% of ARIA-related symptoms resolved completely within the lecanemab and donanemab clinical trials [9, 10, 33].

Severe and serious ARIA is defined by symptoms that completely disrupt daily activity and/or by MRI features of severe ARIA (Table 1) regardless of clinical presentation. If severe ARIA is suspected, Aβ immunotherapy should be discontinued permanently, and an MRI be obtained immediately. Treatment includes intravenous high dose corticosteroids and in serious cases referral to a critical care unit [12–14, 34]. The outcome of severe ARIA varies: Complete resolution of symptoms, permanent neurological disability, and death have been reported [12, 13].

## Conclusion and future directions

The approval of lecanemab and donanemab marks a new era in the treatment of AD. As these medications become more broadly available, clinicians and patients may wish for precise ARIA risk prediction. Multiple risk factors of ARIA have been identified, most notably the APOE4 genotype, baseline cerebral hemorrhage, severe white matter changes, and poorly controlled arterial hypertension, and recommendations for clinical use have been established accordingly. To further specify clinical risk assessment and improve patient safety, the following approaches may be considered in the future:

Antibody selection according to patients’ individual ARIA risk: Since ARIA risk seems to differ between anti-Aβ antibodies, a treatment most compatible with the patient’s risk profile may be chosen. Long-term studies in more diverse patient populations are warranted to compare ARIA risk between different antibodies and guide clinical decision-making in the future.

Modification of dose titration or delivery mode: The modified titration regimen of donanemab with reduced ARIA rates [29] may open up future avenues to be explored, as similar results may be achieved with other antibodies by adjusting titration schemes. Novel possibilities of reducing ARIA risk may also arise from changing antibody delivery mode as exemplified by trontinemab, a modified version of the Aβ fibril-binding antibody gantenerumab using a brain-shuttle technology to cross the blood-brain barrier [49]. Interim data of a phase I study (Brainshuttle AD) indicates that trontinemab clears Aβ from the brain within three months while causing almost no ARIA, possibly due to its unique delivery mode to the brain that reduces direct interactions with vascular Aβ, offering the potential to clear Aβ without increasing ARIA risk [50, 51].

Assessment of pre-treatment Aβ load and disease stage: Associations between ARIA and pre-treatment amyloid load remain conflicting, as the lecanemab phase 3 trial found no association between baseline amyloid status and ARIA incidence, whereas the donanemab phase 3 trial showed a positive correlation [9, 10]. Ongoing and future studies may elucidate if ARIA risk depends on baseline Aβ load and the clinical stage of AD.

Novel biomarkers to determine ARIA risk: The APOE4 allele is thus far the only biomarker of ARIA risk prediction, and while 35–40% of APOE4 homozygotes on lecanemab develop ARIA, the remaining 60–65% do not [9]. Future research is needed to identify biomarkers that, in conjunction with APOE genotyping, increase accuracy of ARIA risk assessment. Potential candidates, based on our current understanding of ARIA pathophysiology, may include inflammatory and neurovascular markers, and baseline levels of neurodegenerative markers in the CSF that could indicate a higher disease burden.

In conclusion, a deeper understanding of ARIA pathophysiology, validation of additional ARIA biomarkers, and acquisition of long-term safety datasets on risk factors in more heterogeneous patient populations may not only lead to increased accuracy of ARIA risk assessment but also novel individualized preventative and therapeutic options.

## Supplementary Information

Supplementary Material 1.

## Data Availability

All data used for this review are included in this published article and its supplementary information files.

## Abbreviations

Aβ: amyloid-β
ABRA: amyloid-β related angiitis
AD: Alzheimer's disease
APOE: Apolipoprotein E
ARIA: Amyloid-related imaging abnormalities
ARIA-E: Amyloid-related imaging abnormalities with edema
ARIA H: Amyloid-related imaging abnormalities with hemorrhage
CAA: Cerebral amyloid angiopathy
CAA-ri: Cerebral amyloid angiopathy - related inflammation
CSF: Cerebrospinal fluid
EMA: European Medicines Agency
EU: European Union
FDA: U.S. Food and Drug Administration
FLAIR: Fluid attenuated inversion recovery
GRE: Gradient recalled echo
iCAA: Inflammatory cerebral amyloid angiopathy
MHRA: Medicines and Healthcare products Regulatory Agency
MRI: Magnetic resonance imaging
UK: United Kingdom
US: United States
PET: Positron emission tomography
SWI: Susceptibility-weighted imaging
