# Supplementary material for: Amyloid-related imaging abnormalities (ARIA) in Alzheimer’s disease: from pathophysiology to individualized risk assessment
Source: Alzheimers Res Ther. 2026 Mar 28;18:79. doi: 10.1186/s13195-026-02022-7 (PMC13063504; doi:10.1186/s13195-026-02022-7)
Supplement: Supplementary file 1 — Supplementary Material 1. [file 13195_2026_2022_MOESM1_ESM.docx]

**Supplement 1: ARIA incidence and PET centiloid changes shown in Figure 3**

|  | **ARIA-E incidence (%)** | **ARIA-H incidence (%)** | **PET centiloid change** | **Data obtained from** |
| --- | --- | --- | --- | --- |
| Aducanumab  (EMERGE, high dose) | 35.0 | .. | -60 | Budd Haeberlein, S., et al., *Two Randomized Phase 3 Studies of Aducanumab in Early Alzheimer's Disease.* J Prev Alzheimers Dis, 2022. 9(2): p. 197-210. |
| Aducanumab  (EMERGE, low dose) | 26.0 | .. | -41 | Budd Haeberlein, S., et al., *Two Randomized Phase 3 Studies of Aducanumab in Early Alzheimer's Disease.* J Prev Alzheimers Dis, 2022. 9(2): p. 197-210. |
| Aducanumab  (ENGAGE, high dose) | 36.0 | .. | -54 | Budd Haeberlein, S., et al., *Two Randomized Phase 3 Studies of Aducanumab in Early Alzheimer's Disease.* J Prev Alzheimers Dis, 2022. 9(2): p. 197-210. |
| Aducanumab  (ENGAGE, low dose) | 26.0 | .. | -38.5 | Budd Haeberlein, S., et al., *Two Randomized Phase 3 Studies of Aducanumab in Early Alzheimer's Disease.* J Prev Alzheimers Dis, 2022. 9(2): p. 197-210. |
| Aducanumab  (EMERGE + ENGAGE, high dose) | .. | 19.1 | 57* | Tampi, R.R., B.P. Forester, and M. Agronin, *Aducanumab: evidence from clinical trial data and controversies.* Drugs Context, 2021. 10.  *Averaged values from EMERGE high dose and ENGAGE high dose (Budd Haeberlein, S., et al.) |
| Aducanumab  (EMERGE + ENGAGE, low dose) | .. | 12.3 | 39.8* | Tampi, R.R., B.P. Forester, and M. Agronin, *Aducanumab: evidence from clinical trial data and controversies.* Drugs Context, 2021. 10.  *Averaged values from EMERGE high dose and ENGAGE high dose (Budd Haeberlein, S., et al.) |
| Gantenerumab  (Graduate I) | 23.9 | 23.7 | -57 | Bateman, R.J., et al., *Two Phase 3 Trials of Gantenerumab in Early Alzheimer's Disease.* N Engl J Med, 2023. 389(20): p. 1862-1876. |
| Gantenerumab  (Graduate II) | 25.8 | 22.0 | -48 | Bateman, R.J., et al., *Two Phase 3 Trials of Gantenerumab in Early Alzheimer's Disease.* N Engl J Med, 2023. 389(20): p. 1862-1876. |
| Lecanemab  (CLARITY, high dose/biweekly) | 12.6 | 16.9 | -55 | van Dyck, C.H., et al., *Lecanemab in Early Alzheimer's Disease.* N Engl J Med, 2023. 388(1): p. 9-21.  Honig, L. S. *et al.* Updated safety results from phase 3 lecanemab study in early Alzheimer's disease. *Alzheimers Res Ther* 16, 105 (2024). |
| Lecanemab  (BAN 2401-201, high dose/biweekly) | 9.9^1^ | 6.2^1^ | -73^2^ | ^1^Swanson, C.J., et al., *A randomized, double-blind, phase 2b proof-of-concept clinical trial in early Alzheimer's disease with lecanemab, an anti-Aβ protofibril antibody.* Alzheimers Res Ther, 2021. 13(1): p. 80.  ^2^McDade, E., et al., *Lecanemab in patients with early Alzheimer's disease: detailed results on biomarker, cognitive, and clinical effects from the randomized and open-label extension of the phase 2 proof-of-concept study.* Alzheimers Res Ther, 2022. 14(1): p. 191. |
| Lecanemab  (BAN 2401-201, high dose/monthly) | 9.9^1^ | 9.5^1^ | -53^2^ | ^1^Swanson, C.J., et al., *A randomized, double-blind, phase 2b proof-of-concept clinical trial in early Alzheimer's disease with lecanemab, an anti-Aβ protofibril antibody.* Alzheimers Res Ther, 2021. 13(1): p. 80.  ^2^McDade, E., et al., *Lecanemab in patients with early Alzheimer's disease: detailed results on biomarker, cognitive, and clinical effects from the randomized and open-label extension of the phase 2 proof-of-concept study.* Alzheimers Res Ther, 2022. 14(1): p. 191. |
| Lecanemab  (BAN 2401-201, intermediate dose/biweekly) | 3.3^1^ | 14.1^1^ | -46^2^ | ^1^Swanson, C.J., et al., *A randomized, double-blind, phase 2b proof-of-concept clinical trial in early Alzheimer's disease with lecanemab, an anti-Aβ protofibril antibody.* Alzheimers Res Ther, 2021. 13(1): p. 80.  ^2^McDade, E., et al., *Lecanemab in patients with early Alzheimer's disease: detailed results on biomarker, cognitive, and clinical effects from the randomized and open-label extension of the phase 2 proof-of-concept study.* Alzheimers Res Ther, 2022. 14(1): p. 191. |
| Lecanemab  (BAN 2401-201, intermediate dose/monthly) | 2.0^1^ | 13.7^1^ | -31^2^ | ^1^Swanson, C.J., et al., *A randomized, double-blind, phase 2b proof-of-concept clinical trial in early Alzheimer's disease with lecanemab, an anti-Aβ protofibril antibody.* Alzheimers Res Ther, 2021. 13(1): p. 80.  ^2^McDade, E., et al., *Lecanemab in patients with early Alzheimer's disease: detailed results on biomarker, cognitive, and clinical effects from the randomized and open-label extension of the phase 2 proof-of-concept study.* Alzheimers Res Ther, 2022. 14(1): p. 191. |
| Lecanemab  (BAN 2401-201, low dose/biweekly) | 1.9^1^ | 5.8^1^ | -22^2^ | ^1^Swanson, C.J., et al., *A randomized, double-blind, phase 2b proof-of-concept clinical trial in early Alzheimer's disease with lecanemab, an anti-Aβ protofibril antibody.* Alzheimers Res Ther, 2021. 13(1): p. 80.  ^2^McDade, E., et al., *Lecanemab in patients with early Alzheimer's disease: detailed results on biomarker, cognitive, and clinical effects from the randomized and open-label extension of the phase 2 proof-of-concept study.* Alzheimers Res Ther, 2022. 14(1): p. 191. |
| Donanemab  (Trailblazer Alz) | 27.5 | 30.5 | -85 | Mintun, M.A., et al., *Donanemab in Early Alzheimer's Disease.* N Engl J Med, 2021. 384(18): p. 1691-1704. |
| Donanemab  (Trailblazer Alz2) | 24.0 | 31.0 | -88 | Sims, J.R., et al., *Donanemab in Early Symptomatic Alzheimer Disease: The TRAILBLAZER-ALZ 2 Randomized Clinical Trial.* Jama, 2023. 330(6): p. 512-527. |
| Bapineuzumab  (NCT00574132, (APOE4 carriers, low dose) | 15.3 | .. | .. | *Salloway S., et al., Bapineuzumab 301 and 302 Clinical Trial Investigators. Two phase 3 trials of bapineuzumab in mild-to-moderate Alzheimer's disease. N Engl J Med. 2014 Jan 23;370(4):322-33.* |
| Bapineuzumab  (NCT00575055, APOE4 non-carriers, low dose) | 4.2 | .. | .. | *Salloway S., et al., Bapineuzumab 301 and 302 Clinical Trial Investigators. Two phase 3 trials of bapineuzumab in mild-to-moderate Alzheimer's disease. N Engl J Med. 2014 Jan 23;370(4):322-33.* |
| Bapineuzumab  (NCT00575055, APOE non-carriers, intermediate dose) | 9.4 | .. | .. | *Salloway S., et al., Bapineuzumab 301 and 302 Clinical Trial Investigators. Two phase 3 trials of bapineuzumab in mild-to-moderate Alzheimer's disease. N Engl J Med. 2014 Jan 23;370(4):322-33.* |
| Bapineuzumab  (NCT00575055, APOE non-carriers, high dose) | 14.2 | .. | .. | *Salloway S., et al., Bapineuzumab 301 and 302 Clinical Trial Investigators. Two phase 3 trials of bapineuzumab in mild-to-moderate Alzheimer's disease. N Engl J Med. 2014 Jan 23;370(4):322-33.* |
| Bapineuzumab  (NCT00676143, APOE4 carriers, low dose) | 15.7 | .. | .. | *Salloway S., et al., Bapineuzumab 301 and 302 Clinical Trial Investigators. Two phase 3 trials of bapineuzumab in mild-to-moderate Alzheimer's disease. N Engl J Med. 2014 Jan 23;370(4):322-33.* |
| Bapineuzumab  (NCT00667810, APOE 4 non-carriers, low dose) | 4.9 | .. | .. | *Salloway S., et al., Bapineuzumab 301 and 302 Clinical Trial Investigators. Two phase 3 trials of bapineuzumab in mild-to-moderate Alzheimer's disease. N Engl J Med. 2014 Jan 23;370(4):322-33.* |
| Bapineuzumab  (NCT00667810, APOE4 non-carriers, intermediate dose) | 11.8 | .. | .. | *Salloway S., et al., Bapineuzumab 301 and 302 Clinical Trial Investigators. Two phase 3 trials of bapineuzumab in mild-to-moderate Alzheimer's disease. N Engl J Med. 2014 Jan 23;370(4):322-33.* |
